# Supplementary material for: The Potential Economic Impact of the Updated COVID-19 mRNA Fall 2023 Vaccines in Japan
Source: Vaccines (Basel). 2024 Apr 18;12(4):434. doi: 10.3390/vaccines12040434 (PMC11054439; doi:10.3390/vaccines12040434)
Supplement: Supplementary file 1 [file vaccines-12-00434-s001.zip › vaccines-2852158-supplementary.pdf]

ADDITIONAL RESULTS TABLES

Table S1. Base-case Quality-Adjusted Life-Years (QALYs) Lost

	Total QALYs Lost			Total QALYs Gained (Moderna Vaccine Vs.)		% Change (Moderna Vaccine Vs.)	
	No Vaccine	Moderna Vaccine	Pfizer Vaccine	No Vaccine	Pfizer Vaccine	No Vaccine	Pfizer Vaccine
Morbidity	1,303,544	1,036,697	1,077,133	266,847	40,436	20.5%	3.8%
Mortality	496,664	289,642	309,998	207,022	20,356	41.7%	6.6%
**Total**	**1,800,209**	**1,326,339**	**1,387,131**	**473,870**	**60,792**	**26.3%**	**4.4%**

Table S2. Base-case Disaggregated Economic Results

Economic Results (Yen, in millions)							
	Total Costs			Cost Difference (Moderna Vaccine Vs.)		% Change (Moderna Vaccine Vs.)	
**Costs:**	**No Vaccine**	**Moderna Vaccine**	**Pfizer Vaccine**	**No Vaccine**	**Pfizer Vaccine**	**No Vaccine**	**Pfizer Vaccine**
Vaccination*	¥0	¥1,120,028	¥1,120,028	¥1,120,028	¥0	--	0%
Adverse Events	¥0	¥3,422	¥3,422	¥3,422	¥0	--	0%
Outpatient Care	¥1,106,588	¥885,165	¥919,240	-¥221,423	-¥34,076	-20%	-4%
Hospitalization	¥663,846	¥399,576	¥425,841	-¥264,270	-¥26,265	-40%	-6%
Infection-Related Myocarditis	¥908	¥692	¥722	-¥216	-¥30	-24%	-4%
**Total (treatment-related)**	**¥1,771,342**	**¥1,285,433**	**¥1,345,803**	**-¥485,909**	**-¥60,370**	**-27%**	**-4%**
**Total (healthcare perspective)**	**¥1,771,342**	**¥2,408,883**	**¥2,469,253**	**¥637,540**	**-¥60,370**	**36%**	**-2%**
Lost productivity	¥1,852,273	¥1,595,264	--	-¥257,009	--	-14%	--
**Total (societal perspective)**	**¥3,623,615**	**¥4,004,147**	**--**	**¥380,532**	**--**	**11%**	**--**

Table S3. Deterministic sensitivity analysis economic results (Moderna Updated Fall 2023 Vaccine relative to No Fall 2023 Vaccine)

		ICER (¥ per QALY Gained)		Change from Base-Case		Change from Base-Case (%)	
**Model Parameter**	**Variation**	**Low Value**	**High Value**	**Low Value**	**High Value**	**Low Value**	**High Value**
Moderna Fall 2023 Vaccine Cost	¥10,072 - ¥18,525	¥1,044,141	¥2,338,205	-¥301,251	¥992,813	-22%	74%
Discount rate	(0%, 4%) per guidelines	¥1,247,145	¥1,428,575	-¥98,246	¥83,183	-7%	6%
Perspective	Societal	¥803,030		-¥542,362		-40%	
Target population	60-64 high-risk and 65+ general	¥911,015		-¥434,377		-32%	
Target population	65+ general	¥942,104		-¥403,288		-30%	
Vaccination strategy	2 booster	¥1,599,586		¥254,194		19%	
Hospitalization rates	See clinical manuscript	¥1,491,297	¥1,173,984	¥145,905	-¥171,408	11%	-13%
Mortality rates	95% CI	¥1,427,202	¥1,264,468	¥81,810	-¥80,924	6%	-6%
Initial VE: hospitalization	Lower and upper bounds from VERSUS	¥2,204,076	¥1,092,674	¥858,684	-¥252,718	64%	-19%
Initial VE: infection	Lower and upper bounds from VERSUS	¥3,843,331	¥539,116	¥2,497,939	-¥806,276	186%	-60%
Vaccine waning: hospitalization	Decreased and increased fall 2023 waning	¥1,223,119	¥1,529,712	-¥122,273	¥184,320	-9%	14%
Vaccine waning: infection	Decreased and increased fall 2023 waning	¥595,046	¥4,274,696	-¥750,346	¥2,929,304	-56%	218%
Vaccine coverage	50%, 75%	¥1,411,804	¥1,397,851	¥66,412	¥52,459	5%	4%
Incidence	Immune Escape April 2024	¥2,051,738		¥706,346		53%	
Incidence	Immune Escape June 2024	¥2,151,514		¥806,122		60%	
Incidence	Adjusted Tokyo Data (2.5x)	¥5,634,508		¥4,289,117		319%	

Incidence	Adjusted Tokyo Data (1.5x)	¥495,259		-¥850,133		-63%	
Incidence	Seasonality: phi=0.2	¥1,205,428		-¥139,964		-10%	
Incidence	Adjusted Tokyo Data (2.0x), Revised Waning	¥2,954,554		¥1,609,162		120%	
Hospitalization cost	+25%, -25%	¥1,205,970	¥1,484,813	-¥139,421	¥139,421	-10%	10%
Percentage with symptoms	95% CI (upper bound, lower bound)	¥1,118,259	¥1,625,364	-¥227,133	¥279,972	-17%	21%
Outpatient cost	+25%, -25%	¥1,228,576	¥1,462,208	-¥116,816	¥116,816	-9%	9%
QALY Losses (non-hospitalized, hospitalized)	+25%, -25%	¥1,178,971	¥1,566,518	-¥166,421	¥221,126	-12%	16%
Percentage in ICU	95% CI (upper bound, lower bound)	¥1,336,406	¥1,354,394	-¥8,986	¥9,002	-1%	1%

A sensitivity analyses was conducted with the unit price of the Moderna updated COVID-19 mRNA Fall 2023 vaccine unit cost. If the unit cost of the vaccine is increased from ¥12,040 to ¥32,513, the incremental cost-effectiveness ratio (ICER) for the Moderna vaccine compared to no vaccine will be ¥5 million per quality-adjusted life-years (QALYs) gained. If the unit cost is increased to ¥39,045 or ¥65,174 the cost per QALY gained will be ¥6 million and ¥10 million respectively.

Table S4. Deterministic sensitivity analysis clinical results (Moderna Updated Fall 2023 Vaccine relative to No Fall 2023 Vaccine)

	Symptomatic infections			Hospitalizations			Deaths		
Scenario	No Vaccine	With Vaccine	Prevented	No Vaccine	With Vaccine	Prevented	No Vaccine	With Vaccine	Prevented
Base case	35,240,923	28,055,308	7,185,614	689,973	417,839	272,133	61,738	36,128	25,610
60-64 high-risk and 65+ general	35,240,923	32,077,805	3,163,118	689,973	484,803	205,170	61,738	40,427	21,311
65+ general	35,240,923	32,375,370	2,865,553	689,973	510,882	179,090	61,738	42,404	19,335
2 booster*	35,240,923	25,549,267	9,691,656	689,973	365,258	324,715	61,738	30,961	30,777
Higher hospitalization rates	35,240,923	28,055,308	7,185,614	799,370	483,249	316,121	71,558	41,868	29,690
Lower hospitalization rates	35,240,923	28,055,308	7,185,614	607,756	368,653	239,103	54,320	31,792	22,528
Higher mortality rate	35,240,923	28,055,308	7,185,614	689,973	417,839	272,133	68,072	39,831	28,241
Lower mortality rate	35,240,923	28,055,308	7,185,614	689,973	417,839	272,133	55,920	32,734	23,186
Low hospital initial VE	35,240,923	28,055,308	7,185,614	689,973	569,299	120,673	61,738	51,058	10,680
High hospital initial VE	35,240,923	28,055,308	7,185,614	689,973	351,576	338,397	61,738	29,597	32,141
Low infection initial VE	35,240,923	33,484,368	1,756,555	689,973	482,140	207,833	61,738	42,242	19,496
High infection initial VE	35,240,923	22,582,279	12,658,644	689,973	343,660	346,313	61,738	29,291	32,447
Low waning (infection)	35,240,923	23,163,057	12,077,866	689,973	350,508	339,465	61,738	29,973	31,765
High waning (infection)	35,240,923	33,776,540	1,464,382	689,973	492,991	196,982	61,738	43,129	18,609
Low waning (hospitalization)	35,240,923	28,055,308	7,185,614	689,973	387,503	302,470	61,738	33,137	28,601

High waning (hospitalization)	35,240,923	28,055,308	7,185,614	689,973	458,419	231,554	61,738	40,130	21,608
Vaccine Coverage (75% of base case)	35,240,923	30,083,501	5,157,422	689,973	486,125	203,848	61,738	42,449	19,289
Vaccine Coverage (50% of base case)	35,240,923	31,859,918	3,381,005	689,973	553,622	136,350	61,738	48,771	12,968
Immune Escape April 2024	43,148,725	38,087,118	5,061,607	842,573	612,108	230,465	74,951	53,768	21,183
Immune Escape June 2024	39,068,442	34,279,489	4,788,953	759,749	532,840	226,909	67,622	46,392	21,230
Adjusted Tokyo Data (2.5x)	33,985,577	32,520,032	1,465,545	566,310	420,523	145,788	51,468	37,864	13,604
Adjusted Tokyo Data (1.5x)	33,606,420	22,216,834	11,389,586	829,881	408,036	421,845	72,775	34,126	38,648
Seasonality: phi=0.2	36,426,098	28,859,505	7,566,593	723,775	432,012	291,762	65,096	37,601	27,496
Adjusted Tokyo Data (2.0x), Revised Waning*	39,927,629	38,219,091	1,708,538	888,266	612,731	275,534	78,996	53,710	25,286
Low percentage with symptoms	31,534,370	25,104,521	6,429,849	617,403	373,892	243,511	55,245	32,329	22,916
High percentage with symptoms	38,942,262	31,001,946	7,940,316	762,440	461,725	300,715	68,222	39,923	28,299

*** 2 Booster Strategy: One dose is offered to adults aged 18 years and older in Fall 2023; A second booster dose is offered to high risk individuals aged 18 – 64 and all individuals ages 65 years and older in Spring 2024.**

Table S5. Economically Justifiable Price Difference* (Moderna Price Difference vs. Pfizer-BioNTech)

	**Moderna Price Difference vs. Pfizer-BioNTech†**		
**Scenario**	**¥5 mil WTP**	**¥6 mil WTP**	**¥10 mil WTP**
Base-case	¥5,022	¥5,860	¥9,212
Relative vaccine effectiveness lower bound	¥2,829	¥3,303	¥5,199
Relative vaccine effectiveness upper bound	¥7,480	¥8,726	¥13,709

†Vaccine administration cost is equivalent for Moderna and Pfizer vaccines, so estimates reflect the economically justifiable price difference

* The price difference between Moderna and Pfizer-BioNTech is the same regardless of the unit cost of the Moderna vaccine.
